# A Unique Population: Adipose-Resident Regulatory T Cells

**DOI:** 10.3389/fimmu.2018.02075

**Published:** 2018-09-28

**Authors:** Qin Zeng, Xiaoxiao Sun, Liuling Xiao, Zhiguo Xie, Maria Bettini, Tuo Deng

**Affiliations:** ^1^Department of Metabolism and Endocrinology, The Second Xiangya Hospital, Central South University, Changsha, China; ^2^Key Laboratory of Diabetes Immunology, Central South University, Ministry of Education, Changsha, China; ^3^Center for Bioenergetics, Weill Cornell Medical College, Houston Methodist Research Institute, Houston, TX, United States; ^4^Section of Diabetes and Endocrinology, Department of Pediatrics, Baylor College of Medicine, McNair Medical Institute, Texas Children's Hospital, Houston, TX, United States

**Keywords:** adipose tissue, inflammation, regulatory T cell, obesity, metabolic disease

## Abstract

Regulatory T (Treg) cell is well known for its anti-inflammatory function in a variety of tissues in health and disease. Accordingly, Treg cells that reside in adipose tissue exhibit specific phenotypes. Their numbers are regulated by age, gender and environmental factors, such as diet and cold stimulation. Adipose-resident Treg cells have been suggested to be critical regulators of immune and metabolic microenvironment in adipose tissue, as well as involved in pathogenesis of obesity-related metabolic disorders. This review surveys existing information on adipose-resident Treg cells. We first describe the origin, phenotype and function of adipose-resident Treg cells. We then describe the major regulators of adipose-resident Treg cells, and discuss how the adipose-resident Treg cells are regulated in lean and obese conditions, especially in humans. Finally, we highlight their therapeutic potential in obesity-related disorders.

## Introduction

Adipose tissue inflammation is implicated in associations between obesity and its multiple complications (1, 2). Obese adipose tissue is laden with a variety of pro-inflammatory immune cells, including classically activated macrophages, natural killer (NK) cells, mast cells, neutrophils, dendritic cells (DCs), B cells, cytotoxic T cells and Th1 cells (3, 4). These cells can stimulate adipocytes and release pro-inflammatory factors, such as IFNγ, TNFα, IL-1β, and IL-6, leading to the development of local and systemic inflammation, insulin resistance, and type 2 diabetes. In contrast, lean adipose tissue contains significantly lower numbers of immune cells, the majority of which are anti-inflammatory cells including Treg cells, alternatively activated macrophages, eosinophils, and group 2 innate lymphoid cells (ILC2s). These cells actively maintain metabolic homeostasis in adipose tissue by secretion of anti-inflammatory mediators, such as IL-4 and IL-13.

An important anti-inflammatory immune cell population in adipose tissue is Treg cell. Treg cells play critical roles in the regulation of autoimmunity (5), allergy (6), microbial infection (7), and tumor development (8). Besides the classical Treg cells circulating through lymphoid organs, Treg cells residing in parenchymal tissue, so-called tissue Treg cells, are also crucial for the maintenance of organismal homeostasis (9). Adipose-resident Treg is one of the best-characterized tissue Treg cells. Dr. Diane Mathis's group published pioneering work in the field of adipose-resident Treg cells. In 2009, they reported that the abdominal fat of lean mice was enriched in Treg cells with a unique phenotype, and these adipose-resident Treg cells played an important role in maintaining insulin sensitivity through limiting adipose tissue inflammation. Importantly, these cells decreased in fat tissue of obese animals (10). Three years later, the same group reported the molecular basis of this unique population of visceral adipose tissue (VAT) Treg cells by identifying peroxisome-proliferator-activated receptor γ (PPARγ), the master regulator of adipocyte differentiation, as the major driver behind development, tissue accumulation, and functional phenotype of these cells (11). Since then, the development, regulation and function of adipose-resident Treg cells have been extensively investigated. Accumulating evidence shows that adipose-resident Treg cells have unique properties, distinct from lymphoid organ Treg cells, and respond to different environmental challenges to regulate immune and metabolic states in normal and disease conditions.

In this review, we summarize current understanding of the characteristics, regulation and function of adipose-resident Treg cells, as well as discuss their metabolic impact and therapeutic potential. Elucidating the interactions underlying adipose-resident Treg cells and adipose inflammation is of significant interest and may provide new insights to overcome the harmful side effects of an obese state.

## Characteristics of adipose-resident Treg cells

Adipose tissue types can be subdivided based on their distinct developmental origins, anatomical locations and functions. These include VAT, subcutaneous adipose tissue (SAT) and brown adipose tissue (BAT). VAT and SAT are composed primarily of white adipocytes with their major function being energy storage, while BAT consists of brown adipocytes with a primary function of thermogenesis. In lean adult male mice, high enrichment of Treg cells (~50% of CD4^+^ T cells) is observed only in VAT, but not in other fat depots (10). Therefore, initial studies were focused on VAT adipose-resident Treg cells. Recently, it has been shown that SAT and BAT also harbor distinct subsets of Treg cells characterized by unique gene signatures (12, 13). It is clear that Treg cells in different types of adipose tissue have distinct properties that are critical for the regulation of local immune and metabolic environment. In this section, we review the origin, phenotypes and functions of the Treg cells from different fat depots.

### Origin

In 10-week-old mice VAT and SAT have relatively low frequencies of Treg cells (10–15% of CD4^+^ T cells), comparable to what is observed in lymphoid tissues. However, only VAT Treg cells accumulate overtime reaching 40–50% at around 25 weeks of age (10, 14). The striking enrichment of Treg cells in VAT has attracted significant interest to investigate the origin and development of VAT Treg cells. Classical Treg cells are generated in the thymus or converted from conventional CD4^+^ T cells in the periphery (15, 16). Because the T cell receptor (TCR) repertoire of VAT Treg cells is distinct from VAT conventional T (Tconv) cells, it is unlikely that the Foxp3^+^ Treg cells in the VAT are differentiated from local Tconv cells (10). A recent study further excluded the possibility of conversion of VAT Treg cells from Tconv cells, and also showed little contribution of circulating Treg cells to the VAT Treg cell accumulation (17). This study revealed that VAT Treg cells were mostly seeded from the thymus in the early weeks of life and proliferated in response to antigen(s) presented by major histocompatibility complex class II (MHCII) and IL-33 beyond 10 weeks of age (17). More recent work has further demonstrated the accumulation of Treg cells in VAT was divided into two steps. Firstly, thymus-derived Treg cells were initially induced to express low level of PPARγ and weak VAT-Treg signature within the spleen; secondly, these PPARγ^low^ Treg cells were installed in VAT, where complete VAT-Treg signature was induced by TCR:MHCII interaction, IL-33, and Foxp3 expression. The combination of these consecutive cues was necessary for complete differentiation into the PPARγ^high^ VAT Treg lineage (18).

Origin of Treg cells in SAT and BAT have not been well studied. Since both of these tissues have normal frequencies of Treg cells (about 10% of CD4^+^ T cells) and naïve CD4^+^ T cells from these two tissues produce more induced Treg cells than VAT (13), the SAT and BAT Treg cells probably have different origin from VAT Treg cells.

### Phenotype

The majority of studies addressing the phenotype of adipose-resident Treg cells were performed in VAT. Microarray analysis comparing Treg cells from VAT and lymphoid tissues revealed notable distinction among Treg cells from different tissue sites and showed a unique transcriptional profile in VAT Treg cells. VAT Treg cells displayed a distinct chemokine/chemokine receptor pattern, overexpression of Gm1960 (an IL-10-inducible Cxcr2 ligand), Ccr1, Ccr2, Ccr9, Ccl6, integrin αV, activated leukocyte cell adhesion molecule (Alcam), Cxcl2 and Cxcl10 and reduced expression of Ccl5 and Cxcr3, which might be responsible for their specific local accumulation (10). VAT Treg cells also produced significantly higher levels of IL-10 and showed enhanced IL-10 signaling compared with Treg cells from lymphoid tissue (10). Most VAT Treg cell signature genes were regulated directly or indirectly by PPARγ, a nuclear receptor overexpressed in Treg cells specifically from VAT but not from other tissues (11). Genetic ablation of PPARγ in Treg cells reduced the number and changed the transcriptional signature specifically in VAT Treg cells, indicating that PPARγ is a master regulator defining VAT Treg cell phenotype (11). In an effort to identify the cytokine receptors preferentially expressed by VAT Treg cells, Vasanthakumar et al. found that ST2, the receptor of IL-33, was highly expressed in Treg cells from VAT and was activated by IL-33 to provide a signal that was essential for the development and maintenance of VAT Treg cells (19). Interestingly, in spite of their unique gene expression signatures, VAT Treg cells were similar in their suppressive capacity as spleen Treg cells based on a standard *in-vitro* suppression assay (10). However, it is not clear if there is a difference in *in-vivo* activity between Treg cells from VAT and other tissues.

In addition to the transcriptional signatures that encompass chemokine receptor, transcription factor, and cytokine receptor phenotypes, VAT Treg cells express tissue-antigen specific TCR repertoire. According to results from complementarity-determining region 3α (CDR3α) sequences, VAT Treg cells have a highly restricted distribution of sequences and exhibit distinguishable TCR repertoires from that of their counterparts in the spleen and lymph nodes (10). Furthermore, in Vα2-Vβ4 VAT-Treg TCR transgenic mice frequency and number of total Treg cells are specifically elevated in VAT, but not in the spleen (18). Moreover, VAT Treg cells depend on recognition of antigen(s) presented by MHCII on antigen-presenting cells (APCs) for their retention/accumulation in VAT (17). However, the special antigen(s) recognized by VAT Treg cells remain undiscovered.

Microarray gene expression profiling of BAT Treg cells from C57BL/6 female mice revealed a shared group of signature genes with VAT Treg cells, including PPARγ and IL-10, but also identified a specific BAT Treg gene signature, suggesting a unique subset of Treg cells in BAT (12). Cold exposure changed expression of a very small group of genes in BAT Treg cells, but the majority of genes remained unchanged (12). It is worth noting that this study compared the gene signature of BAT Treg cells from female mice to the previously published gene signature of VAT Treg cells from male mice. The reported BAT Treg-specific gene signature in this study may have been affected by the gender difference. More recently, it has been reported that in young 3-6-week-old mice BAT and SAT harbor higher Foxp3^+^ Treg cell percentages than VAT, and Treg cells in BAT and SAT are more efficiently induced by cold exposure compared to VAT Treg cells (13).

In summary, Treg cells residing in different types of adipose tissue have distinct features, implying their specialized functions in regulation of immune and metabolic homeostasis in and beyond adipose tissue.

### Function

Metabolic disorders are associated with and mediated by inflammatory processes (20, 21). As one of the most potent anti-inflammatory cell types, Treg cells have been proposed to play a protective role in insulin resistance and other metabolic disorders by several gain-of-function experiments (10, 22, 23). In both high-fed diet (HFD)-induced obese mice and mice heterozygous for the yellow spontaneous mutation (Ay/a), injection of IL-2 in complex with IL-2 antibody (mAb) increased the fraction of Treg cells in VAT and spleen, and reduced insulin resistance (10). Oral administration of anti-CD3 antibody and β-glucosylceramide (GC) in leptin-deficient ob/ob mice effectively induced Treg cells and alleviated the metabolic abnormalities, including pancreatic islet cell hyperplasia, fatty liver, adipose tissue inflammation and high blood glucose (23). In addition, adoptive transfer of CD4^+^Foxp3^+^GFP^+^ Treg cells into db/db diabetic mice led to an increase in Foxp3 expression and a decrease in CD8^+^ effector T cells in VAT, as well as a decrease of urinary albumin-to-creatinine ratio and glomerular diameter (22). These observations indicate that Treg cells can not only ameliorate insulin resistance, but also prevent diabetic nephropathy. The above studies used approaches that resulted in global increases of Treg cells, which were not limited to adipose tissue. Therefore, these studies failed to fully clarify the specific contribution of local adipose tissue resident Treg cells to the improvement of metabolic disorders. Unfortunately, an attempt to enhance Treg cells specifically in VAT by transfer of fat-resident Treg cells into obese mice failed due to the lability and low recoverable numbers of VAT Treg cells (10). However, in our recent study, genetic deletion of MHCII in adipocytes of obese mice substantially increased Treg cell fraction specifically in VAT and reduced adipose tissue inflammation and insulin resistance (24). Interestingly, these beneficial effects were dependent on the specific induction of VAT Treg cells, suggesting tissue specific function of VAT Treg cells against obesity-induced adipose inflammation and insulin resistance (24). In line with our observations, VAT-Treg TCR transgenic mice showed significantly improved insulin sensitivity and glucose tolerance (18). Besides adipose inflammation, Hashimoto's Thyroiditis (HT) is also associated with abnormal insulin sensitivity. A recent study focused on HT showed a drop in VAT Treg cells as well as insulin sensitivity in a mouse model of HT. The impaired insulin sensitivity was effectively reversed by the adoptive transfer of CD25^+^Foxp3^+^ Treg cells from peripheral blood; however, subsequent anti-CD25 antibodies administration had no effect on insulin sensitivity. Since anti-CD25 treatment selectively depletes CD25^+^Foxp3^+^ Treg cells in peripheral blood but not in VAT, the finding supports a potential specific role for VAT Treg cells in improving insulin resistance in HT (25).

The contradictory results from loss-of-function experiments are somewhat puzzling. Short term depletion of Treg cells by administration of diphtheria toxin in mice that express diphtheria toxin receptor (DTR) under the control of the Foxp3 promoter (Foxp3-DTR) significantly increased insulin sensitivity in VAT and liver, and decreased fasting insulin levels, but showed marginal *in-vivo* metabolic changes (10). Transgenic mice expressing the Vα5/Vβ8.2 TCR, which was found at a high frequency among obese adipose tissue T cells, displayed a tissue specific decrease of Treg cells in VAT, but did not show any metabolic phenotype in both lean and obese states (26). Similarly, VAT-specific loss of Treg cells by knockout of PPARγ in Foxp3^+^ Treg cells did not affect insulin sensitivity in young, lean and obese mice (11, 27). However, these negatives do not rule out the potential critical role for VAT Treg cells in regulation of metabolism under certain circumstances. In lean condition, a decrease of VAT Treg cells may not change the inflammatory state because of a lack of inflammation in adipose tissue. While in obese condition, where the VAT Treg cells are already dramatically down-regulated and exhibit loss of function, further decrease of VAT Treg cells may not lead to changes in metabolic phenotypes. Interestingly, loss of VAT Treg cells improves glucose homeostasis in aged mice, suggesting its critical role in aging-induced insulin resistance (27). The detailed mechanism underlying the insulin-desensitizing effects of VAT Treg cells in aged mice is unclear and requires further investigation.

SAT and BAT, two important tissues responsible for cold-induced thermogenesis, also harbor tissue resident Treg cells. Cold exposure and beta-adrenergic stimulation enhanced the accumulation of Treg cells in BAT and SAT, suggesting a potential role for adipose-resident Treg cells in cold-induced thermogenesis. Indeed, depletion of Treg cells blunted beta3-adrenergic receptor (ADRB3) agonist-induced expression of thermogenic and lipolytic genes, while adoptive transfer of Treg cells or expansion of Treg cells by IL-2/mAb injection showed opposite effects (13). These results suggested an important role for Treg cells in the regulation of thermogenesis in SAT and BAT. Since specific depletion or expansion of Treg cells in SAT and BAT is currently not possible, it is hard to determine the specific contribution of Treg cells to regulation of metabolic homeostasis, such as body weight and insulin sensitivity, in these tissues.

While additional studies are necessary to clarify the timing and location of the specific functions of adipose-resident Treg cells, it is clear that these cells are important contributors to maintaining immune and metabolic homeostasis, in part through regulation of adipose inflammation, insulin sensitivity, lipolysis and thermogenesis.

## Regulators of adipose-resident Treg cells

### PPARγ

PPARγ is a member of the PPAR subfamily of nuclear hormone receptors. The name PPAR is derived from the first identified member of the family, PPARα, which has the ability to respond to various compounds that induce peroxisome proliferation (28). However, other members of the PPAR family do not show this function; instead, they play important roles in lipid metabolism and metabolic control, especially PPARγ.

PPARγ was first identified by homology cloning in Xenopus, which was reported to stimulate the peroxisomal degradation of fatty acids (29). There are two isoforms of PPARγ, PPARγ1 and PPARγ2, and the latter has additional 30 amino acids at its extreme N terminus in mice (30). Generally, PPARγ is accepted as a master regulator of adipogenesis and transcription of lipogenic genes (31, 32). It is also known as a suppressor of inflammatory signaling and its ability to exert anti-inflammatory effects in both acute and chronic inflammatory diseases (33, 34). PPARγ was reported to be expressed in Treg cells and protected autoimmunity by enhancement of Treg cells (35, 36). Recently, PPARγ was highlighted as a crucial molecular orchestrator of VAT Treg cell accumulation, phenotype and function (11).

Comparing the gene-expression profiles of VAT and lymphoid-organ Treg cells, Cipolletta et al. found that the level of transcripts encoding the nuclear receptor PPARγ was highly expressed in VAT Treg cells (11). Expression of genes that were positively or negatively correlated with PPARγ transcript levels occupied the majority of strongly up- or down-regulated genes in VAT Treg cells compared to lymphoid-tissue Treg cells (11). VAT Treg cells expressed both PPARγ1 and PPARγ2, with a predominance of the former. The evaluation of each isoform's ability to collaborate with Foxp3 to promote the VAT Treg cell gene signature showed that PPARγ1 and PPARγ2 could both induce most of the genes upregulated in VAT Treg cells. However, the difference between the two protein variants was that only PPARγ1 induced suppression of the genes that were down-regulated in VAT Treg cells (11). More importantly, PPARγ is a key controller for the accumulation and phenotype of Treg cells that are present in VAT. Mice lacking PPARγ specifically in Treg cells had lower frequencies and numbers of VAT Treg cells, but did not exhibit any reductions in Treg cells in lymphoid organs. Additionally, PPARγ-mut mice exhibited reduced expression of VAT Treg cell signature genes (11).

Pioglitazone is a well-known insulin-sensitizing agent that improves metabolic indices in obese mice and humans by targeting and activating PPARγ. Pioglitazone treatment induced an impressive enrichment of the fraction and number of Treg cells in epididymal adipose tissue in both lean and obese mice (11). Conversely, a few days of treatment with an irreversible PPARγ inhibitor, GW9662, decreased the fraction of Gata3^+^ Treg cells in VAT, although the fraction and number of total Treg cells in VAT showed no significant differences (11). Since Gata3 expression was reduced in the absence of PPARγ, these observations suggested that PPARγ is important for both development and continuous maintenance of VAT Treg cell phenotype (11).

The transcriptional signature of VAT Treg cells from obese mice showed a strong correlation with the transcriptional changes induced by deficiency of PPARγ, suggesting that obesity might exert its impact on VAT Treg cell through either direct or indirect modulation of PPARγ (14). However, the level of transcripts encoding PPARγ in obese mice was not altered compared with lean mice (14), suggesting that the genes abnormally expressed in the obese state are not associated with a reduced expression of PPARγ. Instead, reduction of PPARγ activity was shown to be mediated through phosphorylation by protein kinase Cdk5 (cyclin-dependent kinase 5), a kinase that is activated in adipocytes of diet-induced obese mice. As a result, Cdk5 mediated phosphorylation of the serine residue at position 273 of PPARγ and led to dysregulation of a large number of genes involved in the pathogenesis of insulin resistance (37). Intriguingly, the transcriptional signature of obese VAT Treg cells was also dependent on PPARγ's phosphorylation of the serine residue at position 273, which was provoked by obesity (14). Therefore, it is likely that the post-translational modification of PPARγ might affect VAT Treg cells in obesity.

### IL-33 and its receptor ST2

IL-33 is a member of the IL-1 family, which includes IL-1α, IL-1β, IL-18, IL-36α, IL-36β, IL-36γ, IL-33, receptor antagonists IL-1Ra, IL-36Ra, IL-38, and anti-inflammatory cytokine IL-37 (38). IL-33 protein is produced mainly by non-hematopoietic cells (39), particularly specialized populations of epithelial and endothelial cells (40, 41). IL-33 was first detected in human lymph node high endothelial venules (42) and it is highly expressed in lymph node and spleen fibroblastic reticular cells in mice and humans (40, 41). In contrast to humans, expression of IL-33 in mouse endothelium seems to be limited to adipose tissue, liver and female reproductive tract (43, 44). ST2 and the shared signaling chain, IL-1RAcP, make up the receptor complex for IL-33. ST2 is thought to be the only functional receptor for IL-33, because systemic inflammation caused by the loss of IL-33 nuclear localization signals or constitutive over-expression of IL-33 was abrogated by deficiency of ST2 (45, 46). ST2 is expressed in many different kinds of cells in VAT, including adipocytes, mast cells, ILC2s, Th2 cells and Treg cells (47).

Many Treg cells in VAT display ST2 and ST2^+^ Treg cell fraction increases with age to achieve nearly 90% of VAT Treg cells in mice at age of 30 weeks, while they are barely detected in the spleen (17). In addition to VAT Treg cells, other tissue-resident Treg cells, like Treg cells in intestine (48) and muscle (9), also show higher percentage of ST2^+^ cells compared with lymphoid organs. It seems that ST2 expression on tissue-resident Treg cells is maintained by IL-33 itself as IL-33 stimulates the expression of ST2 in Treg cells (19).

Accumulating evidence supports the role for IL-33/ST2 axis as a positive regulator of VAT Treg cells by counteracting obesity-induced adipose inflammation and insulin resistance. Mice lacking ST2 or IL-33 that were kept on a normal chow diet had reduced frequencies and numbers of VAT Treg cells (17, 19, 49), while injection of exogenous IL-33 into lean or obese mice stimulated an impressive expansion of VAT Treg cell population (17, 19, 50). In obese mice, IL-33 treatment reduced inflammation of VAT, as well as improved the metabolic indices (50, 51). However, a recent study reported that although administration of IL-33 induced VAT Treg cell expansion, it also promoted insulin resistance. Moreover, while blockade of IL-33/ST2 signaling by anti-ST2 antibody reduced VAT Treg cells, at the same time the treatment increased insulin-stimulated glucose uptake, suggesting a potential for detrimental effects of IL-33 signaling in metabolism (27). The reason for this discrepancy is not clear, but could be due to different mouse colonies utilized, husbandry practices and facilities utilized in the studies.

Studies suggest both direct and indirect mechanisms for IL-33 modulation of VAT Treg cells. One study suggested that IL-33 had an indirect influence on VAT Treg cells through activation of ILC2, which led to co-stimulatory interactions between Treg cell ICOS and ILC2 expressed ICOSL (49). Another study demonstrated that IL-33 could also act directly on VAT Treg cells. When ST2 expression was specifically ablated in Treg cells, the number of Treg cells in both VAT and SAT decreased, while there was no significant change in spleen. Furthermore, the frequency and number of ILC2 did not change in VAT of these mice, which excluded the effects of ILC2 on VAT Treg cells. In addition, other studies also showed direct effects of IL-33 on tissue-Treg cells *in vitro*, including Treg cells from VAT and other tissues (19, 48, 52, 53). Vasanthakumar et al. have shown that myeloid differentiation factor MyD88, an adaptor protein within IL-33 signaling pathway, was essential for the development and maintenance of VAT Treg cells (19).

The main source of IL-33 in adipose tissue was thought to be primarily derived from multiple stromal cell populations, such as podoplanin^+^ (Pdpn^+^) stromal cells (54). IL-33 protein levels in VAT increase with age, accompanied with Treg cell accumulation (19, 54). IL-17A derived from γδ T cells has been identified as a key cytokine that can induce IL-33 production in adipose stromal cells and sustain VAT Treg accumulation, which was evidenced by the observation that mice lacking IL-17A or γδ T cells exhibited significant reduction in IL-33 protein and Treg cell frequencies in adipose tissue but not in spleen (54).

### TCR:MHCII interactions and co-stimulation

T cell activation requires MHC-mediated antigen presentation and co-stimulation. TCR:MHCII interaction provides a fundamental signal to sustain and activate CD4^+^ T cells (55). MHCII expression on APCs, especially on adipocytes, is important for the activation of Th1 cells in adipose tissue in obese state (24, 56–58). In lean condition, MHCII-mediated antigen presentation is required for the development and maintenance of VAT Treg cells (17, 19). In MHCII-deficient mice, the number of VAT Treg cells was dramatically decreased and few of the remaining VAT Treg cells expressed Gata3, a typical VAT Treg cell marker (17). In addition, a close association between myeloid cells expressing MHCII and Treg cells in VAT was observed in wild type mice, but not in MHCII deficient mice (17). In another study, VAT Treg cells were found to express higher level of Nur77 and lower level of TCF7 than splenic Treg cells. Since transcription factors Nur77 and TCF7 were up- and down-regulated in response to TCR signaling respectively, these results suggested TCR signals are critical to maintain VAT Treg cells (19). Further studies revealed that TCR signals induced transcriptional regulators BATF and IRF4, and subsequently turned on the expression of PPARγ and ST2, two key regulators of VAT Treg cell phenotype (19). B7 molecules (CD80 and CD86], the most classical T cell co-stimulators, were also involved in reducing HFD-induced inflammation by maintaining the number of Treg cells in adipose tissue, liver and lymphoid tissues (59, 60). CD80/CD86 double knockout (B7 KO) mice showed a reduction of VAT Treg cells (59, 60), likely a result from the systemic defects of Treg cell development in these mice (61).

Myeloid cells, including macrophages and dendritic cells, have been proposed to be the major MHCII^+^ APCs that provide necessary TCR signals to VAT Treg cells (17). Indeed, adipose tissue macrophages (ATMs) have been shown to contribute to induction and proliferation of VAT Treg cells (62). ATMs from normal mice induced PPARγ-high Treg cells while ATMs from obese mice induced PPARγ-low Treg cells. Moreover, depletion of ATMs resulted in the reduction of adipose-resident Treg cells, while adoptive transfer of ATMs showed opposite result *in vivo* (62), strongly suggesting that the enrichment of Treg cells in adipose tissue is at least partly due to interactions with ATMs. However, deficiency of MHCII specifically in myeloid cells did not change the fraction of VAT Treg cells in chow fed lean mice, although the reduction was observed in obese mice (58). Further studies are warranted to identify or confirm APC populations that are essential to sustain Treg cells in VAT.

### Other regulators

In addition to the regulators mentioned above, the adipose-resident Treg cells could be regulated by other factors, including secretory factors [IL-21 (63), IL-2 (64), and insulin (65)] and transcriptional factors [STAT3 (66), Foxp3 (18), and STAT6 (13)]. Increased accumulation of Treg cells was demonstrated in VAT of IL-21 knockout mice in comparison with wild-type mice fed normal chow or HFD (63). Accordingly, IL-21 knockout mice exhibited improved insulin sensitivity and decreased adipose inflammation, compared with wild-type mice when fed on HFD. This phenotype was accompanied by a higher induction of IRF4 in VAT, which was negatively regulated by IL-21 (63). IL-2 plays a critical role in Treg cell survival and function (67). A recent study reported that adipose invariant natural killer T cells regulated the proliferation and function of adipose Treg cell through IL-2 production (64). Treg cells express insulin receptor and high levels of insulin could impair the ability of Treg cells to produce IL-10 via AKT/mTOR signaling pathway, suggesting that insulin acts as a negative regulator of Treg cells in hyperinsulinemic obese mice (65).

STAT3 expression in T cells was shown to play a crucial role in adaptive immunity in VAT, which may contribute to adipose tissue inflammation and insulin resistance. Treg cell percentages in VAT were increased in T cell specific STAT3 knockout mice compared with wild-type mice fed a HFD (66). However, since reduction in VAT Treg cells was correlated with HFD-induced obesity, and mice lacking STAT3 in T cells gained less body and VAT weights when fed HFD, it is hard to exclude the possibility that the increase of VAT Treg cells is a secondary effect of weight loss in these mice.

Foxp3 is a master regulator in the function and development of Treg cells (68). Although the role of Foxp3 in lymphoid organ Treg cells is well studied, its role in VAT Treg cell is less clear. A recent study has demonstrated Foxp3 was not only a marker to distinguish Treg cells and Tconv cells; it was also essential for Treg cell enrichment in VAT. When Foxp3 was transduced into Tconv cells; these cells accumulated in VAT, while control transduced cells did not. Moreover, almost all the Foxp3 transduced Tconv cells expressed PPARγ and ST2, markers normally associated with in VAT Treg cells (18).

Few studies have investigated potential regulators of Treg cells in BAT and SAT. So far STAT6 has been found to play a role in regulation of these cells. STAT6 knockout mice displayed lower BAT and SAT Treg cell frequencies compared with wild-type mice. *In-vitro*, Treg cell induction of naïve T cells isolated from BAT and SAT in STAT6 knockout mice was also significantly blunted. Moreover, STAT6 inhibitor suppressed Treg cell induction from naïve CD4^+^ T cells in inguinal lymph nodes of BALB/c Foxp3-GFP reporter mice (13).

### Regulatory network controlling adipose-resident Treg cells

**Figure 1:**
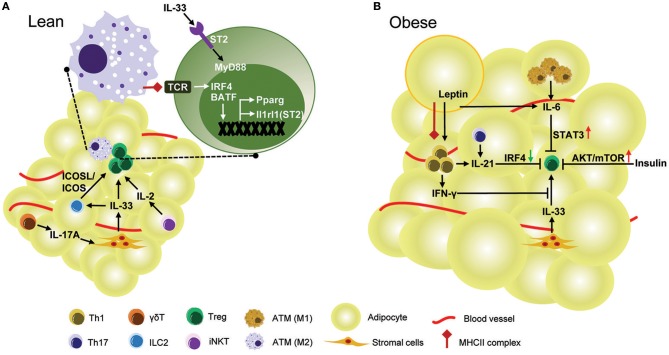
Regulation of Treg cell in mouse VAT. **(A)** In lean mice, healthy adipose tissue is enriched with anti-inflammatory Treg cells. TCR:Antigen recognition and IL-33 signaling are required for the development and accumulation of Treg cells in VAT. The TCR signaling triggered by MHCII complex provided by ATMs up-regulates transcription factors IRF4 and BATF, both of which directly control expression of *Il1rl1* (ST2) and *Pparg* in VAT Treg cells. *Pparg* is the key transcription factor contributing to the unique gene expression profile and phenotype of VAT Treg cells. ST2, encoded by *Il1rl1* gene, is the receptor for IL-33 mediated signaling which promotes proliferation of VAT Treg cells through the adaptor protein MyD88. IL-33 also induces VAT Treg cells indirectly by interactions between Treg ICOS and ILC2 ICOSL. IL-33 is mainly produced by stromal cells and its production is dependent on IL-17A derived from γδ T cells. Regulatory iNKT cells also regulate VAT Treg enrichment by production of IL-2. **(B)** In obese mice, the frequency and number of VAT Treg are drastically reduced. IL-6, which is over-produced by adipocytes and M1-like ATMs in obese mice, suppresses VAT Treg generation by activation of STAT3. IL-21 is overexpressed in obese adipose tissue and reduces VAT Treg cells by down-regulation of IRF4. As obesity develops, adipocytes express more leptin, which in conjuction with MHCII expression by adipocytes, stimulates Th1 cell activation. IFNγ produced by Th1 cells strongly inhibits VAT Treg accumulation by IL-33. Obesity is accompanied by hyperinsulinemia. In obesity, the function of Treg cells is impaired by insulin via AKT/mTOR activation.

Many regulators interact to form a network necessary to maintain proper number and function of adipose resident Treg cells (Figure 1A). PPARγ transcription factor and IL-33/ST2 cytokine/cytokine-receptor pair are key regulators of accumulation and phenotype of VAT Treg cells. ST2 may be regulated by PPARγ, since VAT Treg cells lacking PPARγ show a lower percentage of ST2^+^ cells (17). However, it is likely that PPARγ expression is not affected by IL-33 stimulation (19). Interestingly, both PPARγ and ST2 are direct target genes of transcriptional factor BATF and IRF4, which are induced by TCR signaling (17, 19). Some factors regulate VAT Treg cells indirectly by modulating either the production or function of IL-33. IL-17 secreted by γδ T cells promotes production of IL-33 from stromal cells to sustain VAT Treg cells (54), while production of IFNγ from Th1 cells could inhibit IL-33 signaling to decrease the proliferation of adipose-resident Treg cells (24). Besides IL-33, other cytokines can also act directly on Treg cells in VAT. IL-2, produced by regulatory iNKT cells, can regulates VAT Treg cell enrichment (64). IL-21, production of which is regulated by STAT3 through an autocrine positive-feedback loop (69), negatively regulates IRF4 to decrease Treg cells (63).

## Regulation of adipose-resident treg cells in physiological and pathological processes

In accordance with the important function of adipose-resident Treg cells, they have been described as a highly dynamic population in physiological and pathological processes. A growing number of studies have investigated the regulation of adipose-resident Treg cells under healthy and disease conditions in both humans and mice. These studies help us to better understand the critical role of adipose-resident Treg cells in metabolic diseases, and have identified potential pathways for specific manipulation of adipose-resident Treg cells to improve metabolic homeostasis.

### Age

In lean male mice, VAT Treg cells increase between 5 and 25 weeks of age, then drop rapidly at 40 weeks of age, while splenic Treg cells remain unchanged (10, 14). Consistently, the transcriptional profiles of VAT Treg cells from mice at different ages revealed age-dependent evolution of VAT Treg cell signatures, and demonstrated that numeric changes in VAT Treg cells coincided with functional skewing in aging mice (14). Aging leads to the development of insulin resistance. The decrease of VAT Treg cells with aging is accompanied by a decline in insulin sensitivity, suggesting a protective role of VAT Treg cells in aging-associated insulin resistance (14). Another study showed that the accumulation of VAT Treg cells mainly occurred at age of 4–9 months and declined with further aging at 15 months of age (49). These studies support the idea that development and accumulation of VAT Treg cells are dependent on age and aging leads to the decline in their number. In contrast, a recent study reported a rise of VAT Treg cells in aging mice at age of 44 weeks vs. the young mice at age of 12 weeks (27), suggesting an opposite viewpoint. This discrepancy may be caused by different ways to define control and aging. If the mice at 12 weeks of age are defined as controls, in which the VAT Treg percentage is quite low (~10% of CD4^+^ T cells), we will easily conclude that aging mice (any mice beyond 12 weeks of age) have increased VAT Treg cells. However, if we define the mice at the age of 25 weeks as controls, which are still young and in which the VAT Treg cells reach the peak (~50% of CD4^+^ Tells), we will conclude that aging mice (any mice beyond 25 weeks of age) have decreased VAT Treg cells. Thus, as to the age-dependent VAT Treg cell accumulation, it is likely that all these studies are correct and different controls lead to different conclusions. The investigations on the mechanisms underlying age-dependent VAT Treg cell development and accumulation lead to identification of many key regulators of VAT Treg cells, which were discussed in the above section of “regulators of adipose-resident Treg cells.” The effects of aging on Treg cells in SAT and BAT are still unclear and the data on age-dependent adipose-resident Treg cell accumulation in humans is currently unavailable.

### Cold stimulation

Young lean BALB/c mice (3–6 weeks of age) showed selective accumulation of Treg cells in BAT and SAT, but not in VAT (13). Cold exposure (4°C, 24 h) or beta-adrenergic stimulation increased Treg cell frequencies in all three fat depots; however, accumulation was more efficient in BAT and SAT (13). A T cell-specific STAT6/Pten axis has been suggested to link cold exposure or ADRB3 stimulation with Foxp3^+^ Treg cells induction and adipose tissue function (13). C17orf59, a negative regulator of mTORC1 (70), was induced downstream of STAT6 and mediated Treg cell induction in adipose tissue in response to cold exposure (13). Although the *Adrb3* gene had been reported to be expressed in human lymphocytes (71), it is still unknown if exposure to cold enhances Treg cell induction in human adipose tissue.

### Obesity

Obesity-induced adipose inflammation is in part characterized by the imbalance of local pro-inflammatory T cells and Treg cells, thereby causing obesity-associated metabolic abnormalities. In male mice, it is quite clear that VAT Treg cells are down-regulated in obesity. In a seminal study, Feuerer et al. reported that the frequency and number of VAT Treg cells were dramatically reduced in three mouse models of obesity, including ob/ob mice, Ay/a obese mice, and HFD-induced obese mice, while SAT and splenic Treg cell frequencies were not affected (10). The VAT-specific reduction of Treg cells in obese mice was recently confirmed by another study (14). In addition to reduction in frequencies, the phenotype and function of VAT Treg cells were also influenced by obesity. The transcriptional profiles of VAT Treg cells from either HFD-induced or ob/ob obese mice were significantly different from those of lean mice (14). Some of these differentially expressed genes are important for maintaining the phenotype and function of VAT Treg cells. For example, the expression levels of IL-10 (a key anti-inflammatory cytokine representing Treg cell function) and ST2 (IL-33 receptor, a key receptor defining VAT Treg cells) were substantially reduced in VAT Treg cells, but not in splenic Treg cells isolated from obese mice (19, 65). The transcriptome changes of VAT Treg cells provoked by obesity were thought to be mediated by activation of Cdk5, which deactivated PPARγ through phosphorylation of serine residue 273 (Ser273] (14). While studies in male mice led to the conclusion that obesity decreased the number and changed the transcriptome of VAT Treg cells, the observation did not extend to female mice. In a model of HFD-induced obesity, VAT Treg cells increased proportionally with increase of fat tissue in female, but not in male mice. Accordingly, female mice were better at maintaining metabolic homeostasis, unlike male mice of similar weight, which developed hyperinsulinemia (72). These findings imply that Treg cells can be affected in either a direct or indirect fashion by sexual hormones. Nevertheless, the mechanisms behind the difference in obesity-induced changes of VAT Treg cells between male and female mice are unclear and warrant further investigation. Moreover, the effects of obesity on SAT and BAT Treg cells have not been addressed and remain unknown.

The mechanisms underlying obesity-induced down-regulation of VAT Treg cells in male mice is not fully understood (Figure 1B). IL-21 and STAT3 may be involved in this process. Both IL-21 and STAT3 have been found to be elevated in VAT of mice fed a HFD (63, 66). The mice lacking IL-21 or mice with specific deletion of STAT3 in T cells fed with HFD showed an increase of VAT Treg cells and an improvement of adipose inflammation and insulin sensitivity, suggesting an important role for IL-21 and STAT3 signaling in regulating VAT Treg cells in obesity (63, 66). However, these genetically modified mice were resistant to HFD-induced obesity (63, 66), raising the possibility that the increase of VAT Treg cells is caused by weight loss in these mice, since the abundance of VAT Treg cells is negatively correlated with adiposity in mice. Thus, it is still questionable whether STAT3 and IL-21 have a direct inhibitory effect on VAT Treg cell accumulation in an obese state.

More recently, direct TCR ligation by MHCII expressed by adipocytes was shown to mediate IFNγ production from Th1 cells, which resulted in reduction of VAT Treg cells in obese mice. Obesity increased MHCII expression in adipocytes, while deletion of MHCII specifically in adipocytes (aMHCII^−/−^) led to a decrease of adipose IFNγ expression, an increase of VAT Treg cell abundance and reduced insulin resistance without any changes in weight gain in obese mice. IFNγ treatment effectively blocked IL-33-induced VAT Treg cell proliferation and ST2 expression, whereas HFD-fed IFNγR1^−/−^ mice had similarly high levels of Treg cells in adipose tissue, as did aMHCII^−/−^ mice, and were more insulin sensitive (24). These results suggested a critical role for adipocyte MHCII in the obesity-induced adipose T cell subset switch and insulin resistance.

Although almost all the data from animal models show a decrease in VAT Treg cells in obesity, their regulation by obesity in humans is controversial (Table 1). In most human studies, given that the samples were frozen and cannot be analyzed by flow cytometry, the Foxp3 mRNA levels, presumably an indicator of Treg cells, were determined by quantitative PCR. Consistent to results obtained from mouse models, a human study showed a decrease of Foxp3 gene expression in VAT in obese subjects when compared with lean controls (73). In obese subjects, Foxp3 mRNA levels were higher in SAT than in VAT, and the relative drop in Treg cells in VAT vs. SAT was positively correlated with BMI, suggesting that VAT Treg cells are negatively correlated with obesity in humans (10). In addition, in a cohort of overweight or obese patients, VAT Treg cell number was found to be inversely correlated with plasma fasting glucose and positively correlated with homeostasis model assessment (HOMA)-β (74). These results suggested that consistent with mouse studies, VAT Treg cells in humans are negatively affected by obesity, and have an important role in maintaining glucose. In contrast, several other quantitative PCR-based studies reported an increase of Foxp3 expression and Foxp3/CD3ε ratio in both SAT and VAT in obese subjects (75–77). Frequency of Treg cells measured by flow cytometry analysis was also higher in VAT of obese subjects (78). Moreover, in metabolically healthy obese subjects (MHOS), Foxp3 expression in SAT and VAT were increased as well (79). Analogous to mouse studies, it appears that there is a gender difference in human VAT Treg cell accumulation in obesity. Analysis of CD4^+^CD25^+^Foxp3^+^ or CD8^−^Foxp3^+^ cells showed no difference between overweight and lean female subjects (80). It is important to consider, however, that the inconsistency in results from human studies could be due not only to gender, but also differences in race and age among the cohorts used in the studies. It is also notable that in many studies the Treg cell abundance was indicated by Foxp3 mRNA levels, which is not an ideal indicator of Treg cells because Foxp3 is expressed by a significant proportion of non-Treg cells in humans (81). Additionally, almost all the current human studies recruited limited number of subjects (*N* = 2–44), which may also affect the accuracy of the conclusions drawn. To clarify the regulation of adipose-resident Treg cells in humans, it will be necessary to perform more in-depth analyses on Treg cells from fresh adipose tissue obtained from larger cohorts.

**Table 1:** Change of adipose-resident Treg cells in adipose inflammation of human.

**Subject**	**Sex**	**Mean Age (year)**	**Tissue**	**Method**	**Main findings**	**References**
Control subjects (BMI < 30 kg/m^2^, *n* = 7) and obese subjects without overt type 2 diabetes (BMI ≥30 kg/m^2^, *n* = 13)	No data available	No data available	VAT	Real-time quantitative PCR (Foxp3)	Foxp3 gene expression was lower in VAT in obese subjects compared to control subjects.	(73)
Obese subjects (BMI: 30 to 39.9 kg/m^2^, *n* = 2) and morbidly obese subjects (BMI >40 kg/m^2^, *n* = 9)	Male and female	No data available	VAT and SAT	Real-time quantitative PCR (Foxp3)	The frequency of Treg cells in VAT vs. SAT was negatively correlated with BMI.	(10)
Overweight subjects (BMI ≥25 kg/m^2^, *n* = 44)	Male and female	41.5	VAT and SAT	Flow cytometry	VAT Treg cells were negatively correlated with fasting glucose and MCP-1 and positively correlated with HOMA-β.	(74)
Waist circumferences in lean (<94 cm, *n* = 10), overweight (94–102 cm, *n* = 10) and obese (>102 cm, *n* = 10)	Male	Lean: 43.5, overweight: 48 and obese: 45.2	SAT	Real-time quantitative PCR (Foxp3)	Foxp3 gene expression was higher in SAT in obese subjects compared to control subjects.	(75)
Control subjects (BMI: 17.6 to 26.7 kg/m^2^, *n* = 15) and obese subjects (BMI: 38.7 to 66.0 kg/m^2^, *n* = 36)	Female	Lean: 41.5 and obese: 45.2	VAT and SAT	Real-time quantitative PCR (Foxp3)	Foxp3 gene expression was higher in both VAT and SAT, and the proportion of Treg cells was increased in SAT in obese subjects compared to control subjects	(76)
Control subjects (BMI < 30 kg/m^2^, *n* = 20) and highly obese subjects (BMI >40 kg/m^2^, *n* = 20)	Male and female	No data available	VAT and SAT	Real-time quantitative PCR (Foxp3)	Foxp3 gene expression and the proportion of Treg cells were increased in VAT and SAT in obese subjects compared to control subjects.	(77)
Control subjects (BMI: 18 to 24.9 kg/m^2^, *n* = 16) and obese subjects (BMI ≥30 kg/m^2^, *n* = 15)	Male and female	Lean: 46.07 and obese: 32.83	VAT	Flow cytometry	The frequency of Treg cells was increased in VAT in obese subjects compared to control subjects and the frequency was positively correlated with BMI.	(78)
Control subjects (BMI < 25 kg/m^2^, *n* = 15) and highly obese subjects (BMI >40 kg/m^2^, *n* = 16)	Male and female	27–55	VAT and SAT	Real-time quantitative PCR (Foxp3)	Foxp3 gene expression was increased in both VAT and SAT, and its expression in SAT was positively correlated with weight and BMI, while its expression in VAT was positively correlated with BMI and body fat percentages.	(79)
Control subjects (BMI < 25 kg/m^2^, *n* = 11) and overweight subjects (BMI >25 kg/m^2^, *n* = 15)	Female	38.7	Adipose tissue	Flow cytometry	No difference in the percentage of Treg cells between overweight and control subjects.	(80)

## The therapeutic potential of adipose-resident Treg cells in obesity-associated diseases

Obesity and its related metabolic diseases have been considered as chronic inflammatory diseases. As one of the most potent anti-inflammatory immune cells, Treg cells have been tested as targets for the treatment of many inflammation-related diseases (82). Accumulating evidence shows that modulation of Treg cells, especially VAT Treg cells, represents a potential new strategy for the treatment of obesity-associated diseases (Table 2).

**Table 2:** Therapeutic strategies involving the modulation of adipose-resident Treg cells in metabolic disorders.

**Strategy**	**Subject**	**Mechanism**	**Changes in Metabolic parameters**	**References**
Adoptive Treg cells transfer	db/db mice	Upregulate Foxp3 expression in mVAT; decrease the percentage of pro-inflammatory mVAT-infiltrating CD8^+^CD69^+^ effector T cells	Decrease blood glucose levels and mVAT cell diameter; improve insulin sensitivity	(22)
Injection of mitogenic αCD3 anti-T cell antibody	HFD-fed C57BL/6 mice and ob/ob mice	Restore Treg cell numbers in VAT	Improve glucose tolerance and insulin sensitivity; lose weight transiently	(83)
Injection of the non-mitogenic F(ab′)2 fragment of αCD3	HFD-fed C57BL/6 mice	Restore Treg cell numbers in VAT; increase the MMR^hi^ pool and reduce the MMR^−^ pool; generate an increase in IL-10	Improve glucose tolerance and fasting insulin level	(83)
Oral anti-CD3 in conjunction with oral GC	ob/ob mice	Increase Foxp3^+^ T cells in adipose tissue; decrease CD11b^+^ F4/80^+^ monocytes in adipose tissue	Reduce the level of glucose; reduce pancreatic hyperplasia and hepatic fat accumulation	(23)
Injection of IL-2 and IL-2-specific monoclonal antibody complex	HFD-fed C57BL/6 mice	Increase the fraction of Treg cells in the abdominal fat and spleen	Improve glucose tolerance and insulin sensitivity	(10)
Injection of rIL-33	HFD-fed C57BL/6 mice	Reverse the reduction of Treg cells in obese VAT; reduce VAT inflammation	Reduce fasting glycemia and insulin resistance.	(50)
Treatment with recombinant IL-33	ob/ob mice	Induce accumulation of Th2 cytokines and Th2 cells in WAT; improve differentiation of M2 macrophages in both adipose and liver	Decrease VAT weight and body fat; reduce adipocyte size and blood glucose levels; improve insulin sensitivity	(51)
ADRβ3 stimulation *in vivo* with the β3 receptor agonist CL-316243 (CL)	BALB/c Foxp3-GFP reporter mice	Enhance Foxp3 abundance in CD4 ^+^ T cells from lymph nodes as well as fat tissue	No data available	(13)
Treatment with TUG891	Male mice subjected to SF exposures	Reduce M1/M2 ratios; increase the number of Treg cells in VAT; attenuate VAT inflammation	Reduce food consumption, weight gain and VAT mass; prevent insulin residence	(84)
Treatment with resveratrol (Resv)	Male mice subjected to SF or sleep control conditions	Attenuate the increase of M1 and decrease of M2 induced by SF; abrogate SF-induced reduction in Treg cells; attenuate VAT inflammation	Abrogate the increased fasting insulin and leptin levels associated with SF; attenuate insulin resistance	(85)
Oral Treatment with γ-Aminobutyric Acid	HFD-fed C57BL/6 mice	Increase the frequency of CD4 ^+^ Foxp3 ^+^ Treg cells; reduce the infiltration of macrophage in the adipose tissues	Reduce fasting blood glucose; improve glucose tolerance and insulin sensitivity; inhibit the gain in body weight	(86)
Oral treatment with EPA	C57BL/6/ mice and ob/ob mice	Increase the number of adipose tissue Treg cells	Lower the weight of body and adipose tissues in C57BL/6 mice	(87)
Oral treatment with pioglitazone	HFD-fed C57BL/6 mice	Enrich the fraction and number of Treg cells in epididymal adipose tissue; augment levels of CD36 on the surface of macrophages	Normalize systemic metabolic parameters, including insulin sensitivity and glucose tolerance; increase the level of serum adiponectin	(11)
Oral administration of Akkermansia muciniphila	HFD-fed C57BL/6 mice	Induce Foxp3 Treg cells in the VAT; attenuate adipose tissue inflammation	Improve glucose tolerance; reduce the concentrations of insulin and leptin	(88)
Oral treatment of VAT mixture antigens	HFD-fed C57BL/6 mice	Restore decrease of VAT Treg cells; decrease CD8^+^ T cells infiltration in VAT; limit the switch of M2 to M1 macrophages	Inhibit the gain of body weight and fat mass; improve insulin sensitivity	(89)
Stimulating CD4^+^CD25^−^ cells with the CD3/CD28 antibodies and IL-2/TGF-β	CD4^+^CD25^−^ cells separated from the blood of children with MS or control	Convert conventional CD4^+^CD25^−^ cells into Treg cells *in vitro*	No data available	(90)
Treating isolated Treg cells with EGCG	Treg cells isolated from normal-weight and obese subjects	Enhance the proliferation and IL-10 production of Treg cells *in vitro*; decrease NF-kappaB activity; increase histone deacetylase (HDAC) activity and HDAC-2 expression in Treg cells	No data available	(91)

Adoptive transfer of CD4^+^Foxp3^+^ Treg cells into db/db mice increased Treg cell numbers in VAT and significantly improved insulin sensitivity (22). Other strategies have focused on Treg cell induction *in vivo*. Brief treatment with the mitogenic CD3-specific antibody in HFD-fed C57BL/6 and leptin deficient ob/ob obese mice restored Treg cells numbers in VAT, thereby greatly improving glucose tolerance and insulin sensitivity (83). Consistently, injection of the non-mitogenic F(ab′)2 fragment of CD3-specific antibody into obese mice restored Treg cells in VAT, which was accompanied by a long-term improvement in glucose tolerance (83). Similarly, oral administration of anti-CD3 antibody in conjunction with oral GC in ob/ob obese mice increased Foxp3^+^ T cells and decreased CD11b^+^F4/80^+^ macrophages in adipose tissue, resulting in decreased adipose inflammation and reversal of insulin resistance (23). In addition, expansion of Treg cells in VAT by administration of IL-2 and IL-2-specific monoclonal antibody complex (10) or recombinant IL-33 (50, 51) improved adipose inflammation and insulin resistance in obese mice. All these studies indicated that modulation of VAT Treg cells has a strong potential for treatment of obesity-associated insulin resistance, and provided the rationale to identify new therapeutic targets and develop novel reagents capable of inducing Treg cells in adipose tissues.

*In-vivo* administration of β3 adrenergic receptor agonist CL-316243, which was once used for treatment of obesity and type 2 diabetes (92), enhanced abundance of Foxp3^+^ CD4^+^ T cells in inguinal lymph nodes as well as in adipose tissues, including BAT, SAT, and VAT (13). Other studies found that male mice treated with TUG89, a selective free fatty acid receptor 4 agonist, or resveratrol after being subjected to sleep fragmentation (SF) exposure showed a reversion of SF-associated low M2/M1 ratio, an increase in Treg cell fraction in VAT and improvement of VAT inflammation and insulin resistance (85, 84). Oral administration with γ-aminobutyric acid in HFD-fed mice significantly increased the frequency of Treg cells and reduced infiltration of macrophages in adipose tissues, which correlated with a decrease in fat mass and adipocyte size, improved glucose tolerance and insulin sensitivity (86). Surprisingly, C57BL/6 and ob/ob mice fed with 5% Eicosapentaenoic acid mixed into chow diet had an increase in the number of Treg cells in epididymal adipose tissues compared with control mice fed with normal chow. However, only C57BL/6 mice showed lowered body and adipose tissue weight than controls (87). Recently, it was reported that oral administration of thiazolidinediones (TZDs), PPARγ agonists with insulin-sensitizing function, in HFD-fed mice enhanced the fraction and number of Treg cells specifically in epididymal adipose tissue (11, 27). However, whether the insulin-sensitizing effects of TZDs are dependent on the TZD-induced expansion of VAT Treg cells is still a matter of debate (11, 27).

In addition to conventional medicines, novel strategies have been tested to enhance VAT Treg cells and counteract obesity-related metabolic disorders. A recent study reported that oral administration of Akkermansia muciniphila significantly induced Foxp3 Treg cells in the VAT, subsequently attenuated adipose tissue inflammation and improved glucose tolerance (88). Another study showed that oral treatment of VAT mixture antigens in HFD-fed C57BL/6J mice could not only restore the decrease of VAT Treg cells and reduce the infiltration of CD8^+^ T cells in VAT, but also limit M2-type macrophages changing into M1-type, thereby, reducing the gain of body weight and fat mass, as well as improving insulin sensitivity (89).

Most of the studies discussed so far were carried out in animal models, while only a limited number of studies were performed on human cells or in human subjects. The CD4^+^CD25^−^ cells isolated from the peripheral blood of children with metabolic syndrome (MS) can be converted into Treg cells *in vitro* by the treatment with the CD3/CD28 antibodies and IL-2/TGF-β, suggesting a viable source of functional Treg cells exists in children with MS (90). Another *in vitro* study found that treatment with epigallocatechin gallate (EGCG) enhanced the proliferation and IL-10 production of Treg cells isolated from normal-weight and obese subjects (91). As EGCG is a potent anti-inflammatory agent from green tea, its effects on human Treg cells support an idea that dietary modulation of Treg cells could be a therapeutic strategy for patients with obesity.

Despite great potential benefits of expanding adipose tissue Treg cells, its clinical use is associated with possible risks. The major concern is that excessive Treg cell numbers may suppress the response to infection or promote tumor occurrence (93). Since metabolic diseases are chronic conditions, the treatment options are held to the highest standard for safety. The potential side effects may dampen the enthusiasm for Treg cell therapy in metabolic diseases. The majority of currently available approaches used to expand Treg cells *in vivo* promote systemic increase of Treg cells. Although PPARγ agonist can induce Treg cells specifically in VAT, its unexpected side effects in heart and kidney limit its application. The ideal strategies should include identification of reagents that specifically promote adipose tissue resident Treg cells, or invention of delivery systems specifically targeting adipose tissue.

## Concluding remarks and future outlooks

In recent years, the focus has shifted from lymphoid tissue to tissue resident Treg cells (9, 94). Among them, adipose-resident Treg cell population is one of the best-characterized examples, which displays a unique phenotype. PPARγ has been described to be a key regulator of VAT Treg cell in maintaining its accumulation, phenotype and function. In addition, the proliferation and maintenance of VAT Treg cells are also regulated by other immune cells and cytokines, such as adipose tissue macrophages, iNKT cells, γδT cells, and IL-33. In mice, VAT Treg cells are down-regulated by obesity and may serve as a decisive cell population in the pathogenesis of obesity-related metabolic disorders. Most mouse studies focused on adipose-resident Treg cells support their beneficial function in suppressing inflammation and promoting thermogenesis, suggesting a potential strategy for the treatment of obesity-related metabolic disorders.

However, many questions regarding adipose-resident Treg cell development, tissue accumulation and lineage maintenance still remain to be addressed. Here we highlight several questions that particularly intrigue us and are interesting realms to explore. What antigen(s) are recognized by VAT Treg cells and stimulate their expansion? How do adipose-resident Treg cells acquire the PPARγ activity? Except PPARγ, are there any other regulators that provide adipose-resident Treg cells with unique characteristics? What are the phenotypes and functions of adipose-resident Treg cells in the humans and how are they regulated and modified in human diseases? Finally, how can we specifically manipulate adipose-resident Treg cells? Better understanding of VAT resident Treg cells could be a new strategy to treat or prevent obesity and related metabolic diseases. Studies aimed to address these questions may ultimately represent a potential novel strategy for treating chronic inflammation and metabolic disorders.

## Author contributions

TD conceived the manuscript. QZ, XS, and TD wrote the manuscript. TD, MB, ZX, and LX revised the manuscript.

### Conflict of interest statement

The authors declare that the research was conducted in the absence of any commercial or financial relationships that could be construed as a potential conflict of interest. The handling Editor declared a shared affiliation, though no other collaboration, with one of the authors MB.
